# Special Issue: New Horizon of Plasmonics and Metamaterials

**DOI:** 10.3390/ma13071756

**Published:** 2020-04-09

**Authors:** Shinpei Ogawa, Masafumi Kimata

**Affiliations:** 1 Advanced Technology R&D Center, Mitsubishi Electric Corporation, 8-1-1 Tsukaguchi-Honmachi, Amagasaki, Hyogo 661-8661, Japan; 2 College of Science and Engineering, Ritsumeikan University, 1-1-1 Noji-higashi, Kusatsu, Shiga 525-8577, Japan; kimata@se.ritsumei.ac.jp

**Keywords:** plasmonics, metamaterials, metasurfaces, polarization control, infrared sensors

## Abstract

Plasmonics and metamaterials are growing fields that consistently produce new technologies for controlling electromagnetic waves. Many important advances in both fundamental knowledge and practical applications have been achieved in conjunction with a wide range of materials, structures and wavelengths, from the ultraviolet to the microwave regions of the spectrum. In addition to this remarkable progress across many different fields, much of this research shares many of the same underlying principles, and so significant synergy is expected. This Special Issue introduces the recent advances in plasmonics and metamaterials and discusses various applications, while addressing a wide range of topics in order to explore the new horizons emerging for such research.

Plasmonics and metamaterials are both fields of study that have received increasing attention and that constantly produce new means of modifying electromagnetic waves. Surface plasmon polaritons (SPPs) are electromagnetic waves at the interface between a metal and a dielectric that are excited by the coupling of photons and electrons. In general, SPPs having wavelengths from the ultraviolet to the far infrared (IR) regions of the spectrum can be produced. Metamaterials are engineered artificial structures exhibiting unconventional physical properties that cannot be achieved using standard materials. Recently, there has been significant interest in metasurfaces based on two dimensional metamaterials because of the potential of such surfaces to manipulate photons. These two technologies can be combined, typically in conjunction with optical wavelengths, to produce unique properties. Consequently, many results that are important both in terms of our fundamental understanding of these phenomena and in terms of actual applications have been obtained, using a wide range of materials, structures and wavelengths spanning the ultraviolet to the microwave. Interestingly, despite the numerous fields in which these technologies have been investigated, much of this research has many common principles and so could lead to progress via synergistic effects and collaborations.

This Special Issue, “New Horizon of Plasmonics and Metamaterials”, brings together eight articles and one review that capture and summarize the recent activity and developments in this field as well as practical applications over a wide range of topics, so as to explore the new possibilities emerging for these fields. 

To date, three studies have demonstrated polarization control using metasurfaces or metagratings in the GHz frequency range. Shi et al. [1] employed metasurfaces with multiple layers in association with the generation of vortex beams and conversion via cross-polarization. Shi’s work involved the development of dual metasurfaces for the purpose of polarization conversion as a means of producing beams carrying orbital angular momenta with four different orders (such that l = +1, l = +2, l = −1 and l = −2). The data from this work assisted in the realization of polarimetric and super resolution imaging. Song et al. [2] reported a means of performing the multifunctional manipulation of polarization, based on using a dielectric grating containing periodic arrangements of meta-atoms to produce metagratings. These devices acted as highly efficient waveplates with dual modes and multiple functions, and were able to exhibit a number of different functions. These functions included circular or linear cross-polarization and linear-to-circular polarization conversions as well as mirroring with chirality preservation for a variety of frequency bands, together with significant angular invariance. Such properties could potentially be useful in conjunction with additional ranges of frequencies so as to fabricate small optical polarization control devices for optical, radar and telecommunications applications. Li et al. [3] developed a cross-polarization converter capable of dual-band operation with a transmissive unit cell design with multiple layers, based on a transmit array with aperture coupling. In this device, co-polarized transmittance is greatly reduced (to less than 0.005), giving a ratio of polarization conversion that is almost ideal. This converter has potential applications in telecommunications, radar systems and antennae.

Two studies have examined plasmonic effects in waveguides in conjunction with wavelengths in the visible and near-IR spectral range. Zhang et al. [4] reported a metal-dielectric-metal plasmonic device with gain-assisted operation that exhibited improved slow light operation as a result of a transparency effect related to plasmons. In this system, the optical delay and transmission of slow light can both be improved by adjusting the gain power. In addition, incorporating an additional gain disk cavity allows for the enhanced introduction of slow light via a double-channel. This device could have uses in optical switching, nanolasers and biosensing. Wang et al. [5] produced a metal-insulator-metal (MIM) system incorporating coupled hetero-cavities that generates and tunes three Fano resonances. The multiple Fano resonances are obtained via separate mechanisms involving cavity–cavity coupling and can be considered to represent two different types of resonance. Each type can be tuned separately by modifying various parameters related to the cavities, so as to allow tunable modulation of the Fano resonances. This technology has potential applications in slow-light devices, filters, nanosensors and modulators.

Other work has examined the use of this technology operating at wavelengths at visible and IR wavelengths. Kanamori et al. [6] developed a miniature spectroscope incorporating 25 color sensors in association with Si photodiodes and color filters made of metamaterials. These filters comprised metal gratings exhibiting guided mode resonance, with subwavelength periodic two-dimensional morphologies. The spectral sensitivity of this device was determined to exhibit a peak wavelength that had a linear correlation with the period of the grating. Upon irradiation with monochromatic light at various wavelengths, the incident light’s spectral characteristics could be recovered from the signals generated by the color sensors. This metamaterial filter technology could be applied to the fabrication of image sensors operating in multiple colors. Ogawa et al. [7] researched uncooled IR sensors operating in selective polarization and wavelength capacities to design a means of removing undesirable modes while employing a number of plasmonic metamaterial absorbers (PMAs) and applying a reference pixel and a subtraction process. This same approach has possible applications in a number of different uncooled IR sensors. Ogawa et al. [8] also published a review of the different MIM-PMAs that have been reported. This review discusses the history of these devices together with their basic physical principles and modes of operating, while also addressing the research that will be necessary in the future to elucidate design aspects and allow different applications. The technology discussed by Ogawa could be used in many wavelength regions, including the microwave, terahertz and ultraviolet ranges of the electromagnetic spectrum.

Sabouni-Zawadzka et al. [9] examined a new cellular metamaterial having a simplex tensegrity morphology. Their work involved the use of three different tensegrity lattices with varying geometric structures and assessed six different deformation modes: low and high (double) shear along with soft, stiff and medium extensional. Both unimode and close to bimode lattices were reported, based on a classification system for extreme advanced materials. 

The brief summary above introduces a wide range of topics, including optics, radiofrequency engineering and mechanics. This variety of research illustrates the rapid progress that has occurred in the field of plasmonics and metamaterials. We hope that this Special Issue will inspire researchers to continue to perform groundbreaking research in this area.
